# ChatGPT's Potential in Enhancing Physician Efficiency: A Japanese Case Study

**DOI:** 10.7759/cureus.48235

**Published:** 2023-11-03

**Authors:** Yudai Kaneda, Morihito Takita, Tamae Hamaki, Akihiko Ozaki, Tetsuya Tanimoto

**Affiliations:** 1 Epidemiology and Public Health, School of Medicine, Hokkaido University, Hokkaido, JPN; 2 Internal Medicine, Medical Governance Research Institute, Tokyo, JPN; 3 Internal Medicine, Accessible Rail Medical Services Tetsuikai, Navitas Clinic Shinjuku, Tokyo, JPN; 4 Breast and Thyroid Surgery, Jyoban Hospital of Tokiwa Foundation, Fukushima, JPN; 5 Internal Medicine, Accessible Rail Medical Services Tetsuikai, Navitas Clinic Kawasaki, Kanagawa, JPN

**Keywords:** ai & robotics in healthcare, japan, medical records, differential diagnoses, chatgpt

## Abstract

Artificial intelligence (AI), particularly ChatGPT, developed by OpenAI (San Francisco, CA, USA), is making significant strides in the medical field. In a simulated case study, a 66-year-old Japanese female patient's dialogue with a physician was transcribed and inputted into ChatGPT to assess its efficacy in drafting medical records, formulating differential diagnoses, and establishing treatment plans. The results showed a high similarity between the medical summaries generated by ChatGPT and those of the attending physician. This suggests that ChatGPT has the potential to assist physicians in clinical reasoning and reduce the administrative burden, allowing them to spend more time with patients. However, there are limitations, such as the system's reliance on linguistic data and occasional inaccuracies. Despite its potential, the ethical implications of using patient data and the risk of AI replacing clinicians emphasize the need for continuous evaluation, rigorous oversight, and the establishment of comprehensive guidelines. As AI continues to integrate into healthcare, it is crucial for physicians to ensure that technology complements, rather than replaces, human expertise, with the primary focus remaining on delivering high-quality patient care.

## Introduction

Artificial intelligence (AI), experiencing rapid advancement, is revolutionizing various facets of our lives, including the realm of medicine. Through sophisticated algorithms and machine learning techniques, AI has been instrumental in aiding physicians to diagnose more accurately, pinpoint potential health risks, and tailor personalized treatment plans for patients [1].

One notable AI tool is ChatGPT, developed by OpenAI (San Francisco, CA, USA) and launched on November 30, 2022. Using a method known as reinforcement learning from human feedback, ChatGPT is expected to be a potential assistant in clinical reasoning through linguistic information. Additionally, OpenAI further fortified this potential by releasing the latest iteration of their massive language model, GPT-4, on March 14, 2023. Internal evaluations reveal that compared to its predecessor, the GPT-3.5, the GPT-4 shows an 82% reduction in responding to inappropriate content requests and a 40% increased likelihood of generating factually correct answers [2]. In our empirical examination utilizing the Japanese Nursing National Examination, GPT-3.5's overall accuracy of 59.9% fell short of the passing criteria, while GPT-4 surpassed the threshold with an impressive accuracy rate of 79.7% [3].

With the increasing integration of AI in healthcare, the practical application and adaptability of such models in real-world clinical settings have become paramount. However, considering that ChatGPT was predominantly engineered for interactions in English, including conducting dialogues and dictations, there is a paucity of information regarding its application in Japanese medical settings. To this end, we envisioned a scenario involving a Japanese woman in her 60s presenting with foot swelling following the previous case report [4]. Utilizing a free voice recording tool, we transcribed the patient-physician dialogue and inputted it directly into ChatGPT. Our objective was to assess the efficacy of ChatGPT in drafting medical records, formulating differential diagnoses, and establishing treatment plans in conventional Japanese clinical settings.

## Technical report

The subject of our simulation case scenario is a 66-year-old Japanese female with a medical history of colorectal cancer, diabetes, and obesity. She had been previously identified with an asymptomatic exacerbation of hyperthyroidism following the first dose of the BNT162b2 mRNA COVID-19 vaccine [4]. In September 2023, she revisited us online via Zoom (Zoom Video Communications Inc., San Jose, CA) due to worsening foot edema. During her consultation, the dialogue between the internist and the patient was recorded and transcribed using a free application. This transcript was translated into English using the DeepL Translator (DeepL GmbH, Cologne, Germany), as detailed below in Table 1. We then translated the conversations back into Japanese to ensure that the nuances of the medical conversation were retained.

**Table 1:** Physician-patient conversation on medical history and symptoms

Speaker	Conversation
Physician	I wanted to discuss the increased swelling in your feet you mentioned.
Patient	Yes.
Physician	You mentioned the swelling has been worsening over the past week. Is it more pronounced in the mornings or does it remain consistent throughout the day?
Patient	It tends to get worse by the evening.
Physician	Got it. Does it alleviate a bit in the morning?
Patient	The swelling stays, but doesn't increase in size. However, by evening, marks from my socks remain quite visible.
Physician	Understood. Did this swelling suddenly start or has it been a gradual change over a few months?
Patient	It became more noticeable with the summer heat. I've had a history of heart failure, so I need to stay hydrated. But I've been advised to cut down on salt and water. Since the onset of summer, I've increased my water intake slightly.
Physician	What kind of drinks do you usually have? Like tea or juices?
Patient	Mostly water, sometimes green tea and barley tea.
Physician	I see.
Patient	I have tea quite often.
Physician	You mentioned a history of heart failure. When was the last time you were hospitalized due to that?
Patient	About two years ago, between the end of April and May 2021. I was in for almost a month due to heart failure caused by hyperthyroidism.
Physician	During that time, considering it was hyperthyroidism, did you undergo any cardiac imaging tests?
Patient	My symptoms were quite severe by the time I noticed. I found it hard to walk without getting severely short of breath. I consulted a doctor immediately and...I was admitted right after the consultation.
Physician	That must have been quite unexpected.
Patient	After that, I underwent a series of heart tests.
Physician	Alright. So the cardiac results were fine and the main issue was your hyperthyroidism. You're still on medication to manage your hyperthyroidism, correct?
Patient	Yes, I've been taking methimazole.
Physician	Have you had any recent blood tests for your thyroid function?
Patient	I get them every two months.
Physician	So, as of now, your blood tests are coming back fine?
Patient	Yes. My medication dosage is quite high, almost at its maximum. My doctor has mentioned potentially considering surgery or radiation therapy...
Physician	I see.
Patient	But for now, I'd prefer to stick with the medication and monitor the situation.
Physician	That's understandable. There are several approaches, including optimizing the medication for your thyroid and ensuring stability. However, sometimes it may not be enough with medication alone.
Patient	I understand.
Physician	Regarding the swelling in your feet, do you experience any breathing difficulties when lying down at night?
Patient	No, I don't experience any shortness of breath.
Physician	Thank you. Other than the heart failure, have you been hospitalized or diagnosed with any significant health issues in the past?
Patient	Previously...
Physician	Yes, any past medical history?
Patient	I've had two major surgeries. One in 1997 for a mesenteric mass and the other in 1999 for a rectal issue. I had the latter surgery at the National Cancer Center.
Physician	Thank you, I'm thinking of scheduling a blood test and an X-ray examination.

The entire consultation lasted 7 minutes and 54 seconds, with the transcription in Japanese amounting to 2,167 characters. Subsequently, the above conversation was directly fed into ChatGPT with a directive to create a medical history summary based on it. The instruction provided to ChatGPT was: “As a physician, based on the following conversation, perform clinical reasoning and draft a medical history summary (SOAP format) for this patient.” To ensure patient confidentiality, the mode was set to exclude storing the input as learning data on ChatGPT. Table 2 shows the medical history summaries in the subjective, objective, assessment and plan (SOAP) format by the attending physician and ChatGPT. A high similarity was observed between the content produced by the physician and the content generated by ChatGPT.

**Table 2:** Medical history summaries in SOAP format prepared by the attending physician and ChatGPT SOAP: Subjective, objective, assessment and plan; TSH: Thyroid-stimulating hormone; T3: Triiodothyronine; T4: Thyroxine; fT4: Free thyroxine; PT: Prothrombin time; aPTT: Activated partial thromboplastin time; BNP: Brain natriuretic peptide; BUN: Blood urea nitrogen

SOAP segments	Physician	ChatGPT (GPT-4)
Subjective	Patient reports evening edema and has been focusing on hydration since summer, primarily consuming tea or water. No difficulty in breathing when lying down. Comorbidity: Hyperthyroidism, currently on medication. TSH levels seem to be stable. Past Medical History: Hospitalized previously due to exacerbation of chronic heart failure; history of meningioma and rectal cancer.	Patient reports increased foot swelling over the past week. Swelling intensifies in the evening, with sock indentations persisting into the night. Increased fluid intake due to summer heat. History of hospitalization due to heart failure resulting from hyperthyroidism. Currently on Methimazole, with blood tests conducted every two months. Surgical and radiation treatments are under consideration, but the patient prefers medication management for now. No breathlessness apart from foot swelling. History of two major surgeries: a mesenteric mass removal in 1997 and a rectal surgery in 1999.
Objective	No findings of wheezing on online audio and visual cues.	Observable foot swelling. Methimazole dosage is nearing its maximum due to hyperthyroidism.
Assessment	Differential diagnoses to consider: Hyperthyroidism: Potential overdose of antithyroid medication. Exacerbation of chronic heart failure. Deep vein thrombosis. Renal dysfunction.	Possible recurrence of heart failure: Patient has a history of hospitalization due to heart failure from hyperthyroidism, suggesting potential recurrence of symptoms. Hyperthyroidism: Ongoing treatment with antithyroid medication, nearing maximum dosage, indicates that thyroid status might still be unstable. Renal dysfunction: Foot swelling is a classic symptom. Venous insufficiency: The evening exacerbation of foot swelling suggests this possibility.
Plan	Proceed with the following tests: Blood tests: Complete blood count and general biochemistry, with additional TSH, fT4, PT, aPTT, D-dimer, and BNP. Chest X-ray. Echocardiogram (ECG).	Echocardiogram (ECG): Detailed examination of the heart's functionality and valve conditions to check for signs of heart failure. Thyroid hormone blood test: Measure levels of T3, T4, and TSH to get a detailed assessment of the thyroid function. Renal function tests: Conduct BUN, creatinine, and urinalysis to determine renal status. Doppler ultrasound of the lower limbs: To check for potential circulatory issues in the veins as a cause of the swelling. Lifestyle guidance: Advise on salt intake restriction, appropriate hydration, and elevating the feet during rest.

## Discussion

In this case, we postulated a scenario involving a Japanese female patient in her 60s who presented with worsening edema in her legs. Through this, we aimed to evaluate the potential of integrating ChatGPT as an auxiliary clinical assistant in Japanese medical settings. The medical history summaries generated by both the physician and ChatGPT were largely congruent, underscoring ChatGPT's advanced clinical reasoning capabilities and its prospective role in aiding physicians. Of note, ChatGPT demonstrated the capability to generate a medical history summary of a quality comparable to that of a physician, derived solely from linguistic information obtained through patient interviews, and it accomplished this task within a few seconds. Reportedly, physicians dedicate less than one-third of their clinical time to direct patient interactions, whereas nearly half of their daily consultation duration is consumed by electronic health records and other administrative tasks [5]. By capitalizing on the real-time responsiveness and data processing prowess of ChatGPT, there exists a potential for physicians to devote more time to patient dialogues, thereby substantially curtailing desk work.

Additionally, ChatGPT has demonstrated the capability to systematically generate differential diagnoses with an accuracy exceeding 80% based on linguistic information [6], and recent advancements in software development for auscultation assistance using smartphone audio may further support physicians in making more precise diagnoses, potentially enhancing the efficacy of telemedicine [7]. The integration of such technological innovations holds the potential to pave the way for enhanced operational efficiency for physicians, a reduction in the risk of medical errors, an increase in patient satisfaction, and a consequent improvement in the overall quality of care [8]. Given that ChatGPT was primarily developed for English interactions, conducting dialogues and dictations and giving instructions to ChatGPT entirely in Japanese could have posed challenges. Nonetheless, in this particular instance, ChatGPT offered a cogent response.

While ChatGPT demonstrates promising capabilities, it is crucial to recognize its limitations, especially when it relies solely on linguistic information. In this case, due to the consultation being conducted online via Zoom, there were limited physical objective findings; however, the physician's objective assessment reported no observable wheezing based on the tone of the patient’s voice. This observation, derived from auscultation in the clinical setting, was not verbalized, hence ChatGPT's inability to capture it. When integrating ChatGPT with voice recording applications, it becomes imperative for physicians to articulate their findings during examinations, ensuring they are captured as linguistic data. Furthermore, given the character constraints inherent to ChatGPT, strategies for concise and precise data input are essential to harnessing the system's full potential. Utilizing voice recording applications equipped with features to eliminate redundant filler words during transcription might offer a viable solution to such challenges.

Moreover, physicians must be aware that ChatGPT, while advanced, can produce seemingly accurate but subtly incorrect responses, termed "hallucinations [9]." Furthermore, while its performance is robust in general queries, it falls short in specialized fields like infectious diseases and childcare, failing to meet professional exam standards in these areas [10,11]. Additionally, despite experts acknowledging the ethical concerns of using patient data to inform AI and the risks of clinicians being replaced in medical decision-making processes, there remains a lack of adequate discussion, highlighting the pressing need for system design in the future implementation of ChatGPT in clinical settings [12]. In this context, it is imperative for medical professionals to recognize the skills necessary to harness the capabilities of GPT. There is a need to develop an ecosystem that integrates the improvement and validation of AI performance in medical settings. In this collaborative approach between AI and physicians, it is crucial that physicians take an active and responsible role in overseeing and managing the system to ensure the delivery of high-quality medical care to patients [13].

## Conclusions

Our exploration into the integration of ChatGPT in Japanese clinical settings has revealed its potential as a valuable auxiliary tool with capabilities that can enhance physician efficiency and potentially improve patient care quality. While the system's advanced clinical reasoning and real-time responsiveness offer promising avenues for reducing administrative burdens on physicians, it is not without its limitations. Sporadic occurrences of 'hallucinations' and reduced efficacy in specific domains highlight the need for ongoing assessment and stringent monitoring, underscoring that additional training and adaptation may be required to effectively incorporate ChatGPT into physicians' workflows and mitigate these challenges. As we move forward in this AI-augmented medical landscape, it is paramount for physicians to remain at the forefront, ensuring that technology serves as a complement, not a replacement, and that the primary focus remains on delivering the highest standard of patient care.
